# Effect of Meta-Cognitive Strategy-Based Activities on Executive Functioning, Social Networking, and Instrumental Activities of Daily Living Among Women

**DOI:** 10.7759/cureus.58698

**Published:** 2024-04-21

**Authors:** Punitha P, Jasmine Jessy

**Affiliations:** 1 Occupational Therapy, Saveetha College of Occupational Therapy, Saveetha Institute of Medical and Technical Sciences, Saveetha University, Chennai, IND

**Keywords:** women's health, instrumental activities of daily living, social network, executive function, meta-cognitive strategy

## Abstract

Background

Aging has an impact on women's quality of life and is closely correlated with deteriorating social support systems and cognitive abilities. This project intends to improve women's performance, independence, and quality of life by using meta-cognitive strategy-based activities to improve executive functioning and their capacity to maintain social networks. The ultimate objective is to keep older women's cognitive abilities from declining and to give them more independence in carrying out essential everyday tasks. By enhancing their executive functioning skills, women will be better equipped to navigate daily challenges and maintain their autonomy. Additionally, the project aims to provide a supportive environment for older women to engage in social activities and strengthen their social connections, ultimately leading to a more fulfilling and enriched quality of life.

Aims and objectives

The study aims to evaluate the impact of meta-cognitive strategy-based activities on executive functioning, social networking, and instrumental activities of daily living. This study is specifically for women.

Materials and method

This is a quasi-experimental design with convenience sampling and selected samples (n=70), who were then split into two groups: an experimental group (n=35) and a control group (n=35) based on the inclusion and exclusion criteria. The experimental group received intervention based on meta-cognitive strategies through various activities, while the control group did not receive any specific intervention except health education. Pre- and post-tests were conducted to measure the changes. Executive functioning was assessed using the Brief Executive Function Assessment Tool (BEAT), social networking was assessed using the Lubben Social Networking Scale, and instrumental activities of daily living were assessed using the Lawton Brody Instrumental Activities of Daily Living Scale. The duration of the study was six months, three sessions per week, lasting 45 minutes to an hour each. The statistical analysis was done with significance at a 5% alpha level using IBM SPSS version 29.0 (Armonk, NY: IBM Corp).

Results

The pre-test and post-test data were analyzed using the Wilcoxon signed-rank test and the Mann-Whitney test. Since the p-value of the experimental group was 0.00 for the Lubben Social Networking Scale, the BEAT, and the Lawton Brody Instrumental Activities of Daily Living Scale, the alternate hypothesis was accepted. Thus, the results showed significant improvement in executive functioning, social networking, and instrumental daily living activities in the experimental group compared to the control group.

Conclusion

The clinically significant finding of this study was that the participants were enthusiastic and motivated to engage in meta-cognitive strategy-based activities; furthermore, there was a significant improvement in the experimental group when compared with the control group in executive functioning and social networking abilities. This will enhance functions like instrumental activities of daily living and occupational performance. Meta-cognitive strategy-based activities appeared beneficial for improving the cognitive functions and quality of life of women. Although the findings from the studies are positive for therapeutic and health benefits, more clinical trials are needed in order to prove the effect of meta-cognitive strategy-based activities as a therapeutic approach.

## Introduction

Aging among women can lead to cognitive decline. Studies have shown that women may have greater cognitive reserve but faster cognitive decline compared to men. This decline in cognitive function can be clinically significant and raise the risk of dementia and functional disability [1]. Factors such as age, sex hormones, genetics, and lifestyle factors can contribute to these sex differences in cognitive decline [1]. Aging has a greater impact on cognitive function. The specific cognitive functions that decline with age in women are global cognition and executive function [2]. It was investigated that, as individuals grow older, they may experience declines in memory, attention, and problem-solving skills. Age-related decreases in working memory and processing speed have been strongly associated with lower performance on everyday problem-solving tasks [3]. This is common among older women. Older adults with cognitive complaints tend to report a lower quality of life and more difficulties in daily life. Cognitive decline and everyday functioning, as well as quality of life, are strongly correlated. Cognitive decline is associated with greater depression, anxiety, stress, and impairment in instrumental activities of daily living. These findings suggest that addressing cognitive complaints in older adults may be important for improving their overall well-being [4-6]. Executive functions refer to cognitive processes that are responsible for goal-directed behavior, such as working memory, behavioral inhibition, and task-switching [7]. They play a crucial role in self-regulation and are important for successful self-control and decision-making [8-10]. Executive functions are a family of top-down mental processes that are essential for mental and physical health. Social isolation affects a substantial percentage of adult generations and is a modifiable risk factor. Social isolation can impact mental health. Studies have shown that social isolation is associated with increased risks for depression and anxiety, cognitive health impairment, and reduced daily physical activity. It can also lead to feelings of loneliness and unsatisfactory living sensations [11]. Social networking, or engaging in socializing, has benefited the biological profiles of aging women. Social isolation can have a negative impact on cognitive health in later life [12,3].

Female elderly were more likely to have the risk of limitations in performing activities of daily living and instrumental activities of daily living. Age has an impact on independence in daily life for women. The study found that older age was a determinant of disability in instrumental activities of daily living among older women [13]. Meta-cognition refers to the awareness and understanding of one's own thought processes, while problem-solving skills involve the ability to identify and solve problems. It is mentioned that teaching meta-cognitive strategies can improve task performance by recognizing effective strategy use [14]. It is crucial for women to prioritize their well-being and not hesitate to seek therapy or treatment when needed in order to maintain their overall health and happiness. There is limited research on the relationship between knowledge of meta-cognitive strategies and the use of these strategies. Further investigation is needed to understand the link between executive functions and meta-cognition [15,16]. This research gap highlights the importance of continued study in this area to better support women in maintaining their cognitive health as they age. By addressing these knowledge gaps, researchers can develop more effective interventions and strategies to promote healthy aging for women.

## Materials and methods

The study received ethical approval from the Institutional Scientific Review Board of Saveetha College of Occupational Therapy (Ethical Clearance No.: SCOT/ISRB/019/2023). This research is a quantitative study with a quasi-experimental design. This quasi-experimental study included an experimental group and a control group. This research was conducted in the district of Ranipet, Tamil Nadu, India. Women from various places in this district took part in this study. The sampling technique used was non-randomized convenience sampling. Convenience sampling is useful for obtaining samples and selecting sample representatives from all elements when the population has a wide distribution. The sampling is based on a predetermined population area. First, the sample size of n=70 was determined with reference to a study that incorporated 80 participants [17]. However, 10 participants withdrew due to their personal decisions. Samples were selected based on the inclusion and exclusion criteria. Then they were divided into 35 participants in the experimental group and 35 participants in the control group. Women with poor executive functioning, who are unable to perform instrumental activities of daily living and have poor social networking capability, were included and women with other neurological or cardiorespiratory conditions, dementia, psychiatric conditions, and those involved in other advanced rehabilitation programs were excluded. The experimental group was given meta-cognitive strategy-based activities, which included jump start journal activity, think pair share activity, ball pass activity, and fishbowl activity to facilitate discussion. Then they were given force field analysis activity and photo caption activity, and each one underwent generative knowledge interviewing. To wrap up the figurative transformation activity, index card takeaways were distributed. During the activity, they were encouraged and trained to do self-questioning, self-explanation, and self-assessment. Reviewing and goal setting was done at the end of each session. Simultaneously control group underwent health education, which focused on educating women regarding aging and its effects on holistic health. They were also educated on primary preventive measures that should be taken to avoid dependency and deterioration in quality of life.

The data collection methods used in this research included tests, questionnaires, and documentation. Various assessments are used to evaluate executive functioning, social networking, and instrumental activities of daily living among women. These tests were administered twice (pre-test and post-test). Executive functioning was assessed using the Brief Executive Function Assessment Tool (BEAT) validity of 0.08 reliability of 0.07, social networking was assessed using the Lubben Social Networking Scale validity of 0.9 reliability of 0.8, and instrumental activities of daily living were assessed using the Lawton Brody Instrumental Activities of Daily Living Scale validity of 0.8 reliability of 0.8 [18-20]. The purpose of the study was described to all the participants, and consent to take part in this study was received. From the total of 70 participants, 35 were allotted to the experimental group, and the remaining 35 were assigned to the control group. Both groups underwent a pre-test. Then the experimental group received meta-cognitive strategy-based activities, while the control group received only health education. The duration of the research was six months, and therapy was planned for three times a week. At the end of six months, the control and experimental groups underwent a post-test. Then the results obtained from the tests were analyzed statistically. The statistical analysis was done with significance at a 5% alpha level using IBM SPSS version 29.0 (Armonk, NY: IBM Corp). Wilcoxon signed-rank test and Mann-Whitney U test were used. The pre- and post-test data collected from each scale are compared, and the significance was set at a p-value of 0.05.

## Results

In Table 1, since the p-value of 1.000 is greater than 0.05, the alternate hypothesis is rejected. Hence, there is no statistically significant difference in the pre-test and post-test of the Lubben control group, proving that there is less significant improvement in social networking capacity among those who underwent health education.

**Table 1:** Difference between pre-test and post-test of the Lubben Social Networking Scale in the control group (Wilcoxon signed-rank test) *Significant at 5% (p<0.05). Total range: 0-30; social isolation: <12; higher social network: >12

Test	Mean	N	Z-value	p-value
Pre-test	7.37	35	0	1*
Post-test	7.37	35		

In Table 2, since the p-value of 0.000 is less than 0.05, an alternate hypothesis is accepted. Hence, there is a statistically significant difference between the pre-test and post-test of the Lubben experimental group, proving that there is an improvement in social networking capability among those who underwent meta-cognitive strategy-based activities.

**Table 2:** Difference between pre-test and post-test of the Lubben Social Networking Scale in the experimental group (Wilcoxon signed-rank test) *Significant at 5% (p<0.05). Total range: 0-30; social isolation: <12; higher social network: >12

Test	Mean	N	Z-value	p-value
Pre-test	7.4	35	-4.785	0.000*
					Post-test	18.53	35

In Table 3, since the p-value of 0.000 is less than 0.05, an alternate hypothesis is accepted. Hence, there is a statistically significant difference in post-test scores between the experimental and control groups, proving that the meta-cognitive strategy-based activities were beneficial in improving social networking capacity among women.

**Table 3:** Difference between post-tests of the Lubben Social Networking Scale among the experimental and control groups (Mann-Whitney U) *Significant at 5% (p<0.05). Total range: 0-30; social isolation: <12; higher social network: >12

Test	Mean	N	U-value	p-value
Control	15.5	35	-6.67	0.000*
Experimental	45.5	35		

In Table 4, since the p-value of 1.000 is greater than 0.05, the alternate hypothesis is rejected. Hence, there is no statistically significant difference in the pre-test and post-test of the control group, proving that there is less improvement in executive functioning among those who underwent health education.

**Table 4:** Difference between pre-test and post-test of BEAT in the control group (Wilcoxon signed-rank test) *Significant at 5% (p<0.05). Total range: 0-75; cognitive impairment: <33; good cognitive ability: >33 BEAT, brief executive function assessment tool

Test	Mean	N	Z-value	p-value
Pre-test	35.47	35	0	1*
					Post-test	35.47	35

In Table 5, since the p-value of 0.000 is less than 0.05, an alternate hypothesis is accepted. Hence, there is a statistically significant difference between the pre-test and post-test of the experimental group, proving that there is a significant improvement in executive functioning among those who underwent meta-cognitive strategy-based activities.

**Table 5:** Difference between pre-test and post-test of BEAT in the experimental group (Wilcoxon signed-rank test) *Significant at 5% (p<0.05). Total range: 0-75; cognitive impairment: <33; good cognitive ability: >33 BEAT, brief executive function assessment tool

Test	Mean	N	Z-value	p-value
Pre-test	35.97	35	-4.785	0.000*
					Post-test	52	35

In Table 6, since the p-value of 0.000 is less than 0.05, an alternate hypothesis is accepted. Hence, there is a statistically significant difference in post-test scores between experimental and control groups, proving that there is a significant improvement in executive functioning capacity among those who underwent meta-cognitive strategy-based activities when compared to a control group who underwent health education.

**Table 6:** Difference between post-tests of BEAT among experimental and control groups (Mann-Whitney U) *Significant at 5% (p<0.05). Total range: 0-75; cognitive impairment: <33; good cognitive ability: >33 BEAT, brief executive function assessment tool

Test	Mean	N	U-value	p-value
Control	15.83	35	-6.515	0.000*
					Experimental	45.17	35

In Table 7, since the p-value of 0.317 is greater than 0.05, an alternate hypothesis is rejected. Hence, there is no statistically significant difference in the pre-test and post-test of the control group, proving that there is less improvement in instrumental daily living among those who underwent health education.

**Table 7:** Difference between pre-test and post-test of Lawton Brody Instrumental Activities of Daily Living Scale in the control group (Wilcoxon signed-rank test) Significant at 5% (p<0.05). Total range: 0-8; greater the score=greater the independence

Test	Mean	N	Z-value	p-value
Pre-test	2.63	35	-1	0.317
					Post-test	2.6	35

In Table 8, since the p-value of 0.000 is less than 0.05, an alternate hypothesis is accepted. Hence, there is a statistically significant difference in the pre-test and post-test of the experimental group, proving that there is a significant improvement in instrumental activities of daily living among those who underwent meta-cognitive strategy-based activities.

**Table 8:** Difference between pre-test and post-test of Lawton Brody Instrumental Activities of Daily Living Scale in the experimental group (Wilcoxon signed-rank test) *Significant at 5% (p<0.05). Total range: 0-8; greater the score=greater the independence

Test	Mean	N	Z-value	p-value
Pre-test	3.07	35	-4.859	0.000*
					Post-test	6.6	35

In Table 9, since the p-value of 0.000 is less than 0.05, an alternate hypothesis is accepted. Hence, there is a statistically significant difference in post-test scores between the experimental and control groups, proving that there is significant improvement among those who underwent meta-cognitive strategy-based activities when compared to the control group who underwent health education.

**Table 9:** Difference between pre-test and post-test of Lawton Brody Instrumental Activities of Daily Living Scale among experimental and control groups (Mann-Whitney U) *Significant at 5% (p<0.05). Total range: 0-8; greater the score=greater the independence

Test	Mean	N	U-value	p-value
Control	15.5	35	-6.828	0.000*
					Experimental	45.5	35

## Discussion

This study aimed to determine the effect of activities based on meta-cognitive strategies on women's executive functioning, social networking, and instrumental daily living activities. Data from each scale's pre- and post-tests were compared. Furthermore, the significance was established at p<0. Since the p-value of 0.000 is less than 0.05, an alternate hypothesis is accepted. Hence, there is a statistically significant difference in the pre-test and post-test of the experimental group substantially surpassing the control group in terms of executive function, social networking, and instrumental activities of daily living, according to the results. This suggests that engaging in meta-cognitive strategy-based activities may be a more effective way to improve everyday living skills than undergoing traditional health education sessions. These outcomes demonstrate the potential advantages of using meta-cognitive techniques in interventions meant to enhance daily functioning abilities.

Table 1 shows the difference between the pre-test and post-test of the Lubben Social Networking Scale among the control group. Since the p-value of 1.00 was greater than 0.05, the alternate hypothesis was rejected, and Table 2 shows the results of the pre-test and post-test of the Lubben Social Networking Scale in the experimental group. Since the p-value of 0.000 is less than 0.05, an alternate hypothesis is accepted. Table 3 shows the difference between the post-tests of the control and experimental groups. Since the p-value of 0.000 is less than 0.05, an alternate hypothesis is accepted. Hence, there is a statistically significant difference in post-test scores between the experimental and control groups. These findings are supported by the study, which investigates the effectiveness of meta-cognitive goal-based intervention in improving self-awareness in adults with cognitive-communication disorders who have acquired brain injuries. The intervention, consisting of group therapy, home practice, and goal-based individual therapy, showed significant improvements in emergent awareness and overall self-awareness. The study suggested that meta-cognitive strategy instruction can enhance impaired self-awareness in cognitive communication disorders [21].

Table 4 shows the difference between the pre-test and post-test scores of the control group. Since the p-value of 1.000 is greater than 0.05, the alternate hypothesis is rejected. Hence, there is no statistically significant difference in the pre-test and post-test scores of BEAT in the control group, and Table 5 shows the difference between the pre-test and post-test scores of BEAT in the experimental group. Since the p-value of 0.000 is less than 0.05, an alternate hypothesis is accepted. Table 6 shows the difference in the BEAT between the post-tests of control and experimental. Since the p-value of 0.000 is less than 0.05, an alternate hypothesis is accepted. Hence, there is a statistically significant difference in post-test scores between the experimental and control groups, and there is a significant improvement in executive functioning in the experimental group when compared to the control group. These results were supported by various other researchers conducted by [10,15,16].

Table 7 shows the difference between the pre-test and post-test scores of the Lawton Brody instrumental activities of daily living scale among the control group. Since the p-value of 0.317 is greater than 0.01, an alternate hypothesis is rejected. Table 8 shows the difference between the pre-test and post-test scores of the Lawton Brody Instrumental Activities of Daily Living Scale among the experimental group. Since the p-value of 0.000 is less than 0.01, an alternate hypothesis is accepted. Table 9 shows the difference between the post-scores of the Lawton Brody Instrumental Activities of Daily Living Scale among the experimental and control groups. Since the p-value of 0.000 is less than 0.01, an alternate hypothesis is accepted. Hence, there is a statistically significant difference in post-test scores between the experimental and control groups, suggesting that there is a statistically significant improvement in instrumental activities of daily living among the experimental group when compared with the control group. Additionally, the findings of the study are also supported by a pilot study that aimed to determine if a meta-cognitive, goal-based intervention could help adults with traumatic brain injury maintain and enhance their social communication abilities. Eight participants completed three phases: baseline, eight-week intervention, and follow-up. Additional investigation also supports this finding [22-24]. A study reveals that executive function evaluations are rarely used in post-stroke care. A review of the literature found 17 performance-based measures of executive function and 41 studies on their psychometric qualities, focusing on stroke. The results led to the creation of a toolkit for stroke-specific executive function assessment. The multiple errands test, assessment of motor and process skills, and executive function performance test were found to be the most valid and reliable. The kettle test was found to be the most clinically useful [25].

According to Nelson and Narens' (1990) model of meta-cognition, the meta-level, or meta-cognition, needs to continuously monitor and update the performance of the ongoing object level for evaluation and necessary manipulation in order to efficiently control object-level cognitive processing. Meanwhile, a strong updating skill is required to maintain and update the goals of both processes since monitoring is typically carried out concurrently with object-level processing. Since problem-solving skills are linked to meta-cognition and working memory is a key predictor of thinking and problem-solving abilities, the multi-level model can also be used to explain the significant role that updating plays in meta-cognition [26-29]. However, it is suggested that the Tower of Hanoi puzzle can be used to access the executive functions of inhibition [30]. It is thought that the Tower of Hanoi can access more executive functions than just the basic ones. It also demonstrates planning ability because it entails mentally outlining a course of action before acting. A strategy is required in order to carry out the meticulous planning required for this task's successful completion.

We acknowledge that the limited generalization of our findings is a result of the small sample size and homogeneous sample. Future research ought to make use of bigger and more varied sample sizes. It is well known that the development of executive functions varies. Results from samples with more age and executive function variability may differ from those from our small samples. In order to enable a more reliable test of the relationships between constructs, large samples would also offer the chance to gather multiple measures of each construct under consideration and to use more sophisticated statistical techniques, like structural equation modeling. Future research should focus on more diverse samples and large sample sizes to replicate and extend our findings.

## Conclusions

The most clinically significant finding of the study was that the participants were enthusiastic and motivated to engage in meta-cognitive strategy-based activities; furthermore, there was a significant improvement in the experimental group when compared with the control group in executive functioning and social networking abilities. This improvement will further improve functions like instrumental activities of daily living and occupational performance. Meta-cognitive strategy-based activities appear beneficial for improving the cognitive functions and quality of life of women. Although the findings from the studies are mostly positive for therapeutic and health benefits, more clinical trials are needed to assess meta-cognitive strategy-based activities as a therapeutic approach.
